# Effect of bacterial DNA enrichment on detection and quantification of bacteria in an infected tissue model by metagenomic next-generation sequencing

**DOI:** 10.1038/s43705-022-00208-2

**Published:** 2022-12-26

**Authors:** Vladimir Lazarevic, Nadia Gaïa, Myriam Girard, Florian Mauffrey, Etienne Ruppé, Jacques Schrenzel

**Affiliations:** 1 Genomic Research Laboratory, Division of Infectious Diseases, Department of Medicine, University Hospitals and University of Geneva, Geneva, Switzerland; 2 Université Sorbonne Paris Nord and INSERM UMR1137 IAME, Université de Paris Cité, Paris, France; 3 AP-HP, Hôpital Bichat, Laboratoire de Bactériologie, Paris, France; 4 Bacteriology Laboratory, Division of Laboratory Medicine, Department of Diagnostics, Geneva University Hospitals, Geneva, Switzerland

**Keywords:** Metagenomics, Bacteriology

## Abstract

Before implementing metagenomic next-generation sequencing (mNGS) in the routine diagnostic laboratory, several challenges need to be resolved. To address strengths and limitations of mNGS in bacterial detection and quantification in samples with overwhelming host DNA abundance, we used the pig muscle tissue spiked with a home-made bacterial mock community, consisting of four species from different phyla. From the spiked tissue, we extracted DNA using: (i) a procedure based on mechanical/chemical lysis (no bacterial DNA enrichment); (ii) the Ultra-Deep Microbiome Prep (Molzym) kit for bacterial DNA enrichment; and (iii) the same enrichment kit but replacing the original proteinase K treatment for tissue solubilization by a collagenases/thermolysin digestion and cell filtration. Following mNGS, we determined bacterial: ‘host’ read ratios and taxonomic abundance profiles. We calculated the load of each mock-community member by combining its read counts with read counts and microscopically-determined cell counts of other co-spiked bacteria. In unenriched samples, bacterial quantification and taxonomic profiling were fairly accurate but at the expense of the sensitivity of detection. The removal of ‘host’ DNA by the modified enrichment protocol substantially improved bacterial detection in comparison to the other two extraction procedures and generated less distorted taxonomic profiles as compared to the original enrichment protocol.

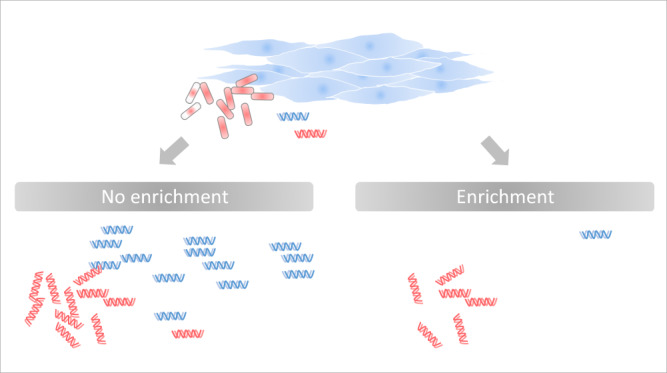

## Introduction

There is a growing clinical interest for the use of metagenomic next-generation sequencing (mNGS) in the diagnosis of infections. mNGS may not only allow faster pathogen identification than standard culture but also may be used to type bacteria and to predict their antibiotic resistance profile, helping to initiate timely and appropriate antibiotic regimen [1, 2]. As an open-ended approach, mNGS does not focus on specific pathogens, but rather captures genetic information from a panoply of bacterial (and other) species present in the sample, including those genetically different from known and common infectious agents. It also detects fastidious and yet-uncultivable bacteria.

In clinical microbiology, mNGS is currently mainly used as a last resort method when routine techniques for pathogen identification fail, but has the potential to eventually overcome many limitations of culture-based, molecular and serological approaches, providing an all-in-one solution [2, 3].

However, the implementation of mNGS in the clinical diagnostic routine requires addressing several challenges including (i) *removal of host DNA* to increase pathogen-to-host signal ratio [4]; *pathogens quantification* in absolute terms; (iii) *identification of contaminants* that originate from reagents, co-processed samples, investigators or laboratory environment [5] and, (iv) *setting up of a reliable bioinformatics pipeline* to provide biologically and clinically meaningful interpretation of the NGS data. Failure in any of these tasks carries the risk of generating erroneous interpretations that may lead to inadequate patient management such as insufficient or unnecessary treatments. In this study, we addressed the first two of the four above-mentioned challenges.

Clinical specimens of infected tissues contain both human and microbial cells. Given the size of the human genome (that equals roughly 1000 bacterial genomes), sequencing of extracted DNA may result in very low proportions of bacterial reads or even erroneous conclusion of the absence of bacteria. Human DNA removal allows deeper exploration of the microbiota by increasing the DNA sequencing bandwidth for microorganisms [2, 6]. The procedures for bacterial DNA enrichment by host DNA depletion include (i) selective lysis of human cells, followed by degradation of released human DNA prior to bacterial lysis and DNA extraction [4] or (ii) post-extraction removal of human DNA based on differential methylation density between prokaryotic and eukaryotic DNA [7].

Following the analysis of NGS data of clinical samples, the reported number of sequencing reads and/or the proportion of reads assigned to a pathogen do not have direct clinical value and should be translated into clinically relevant information. The qPCR targeting the bacterial 16S rRNA gene marker provides a rough estimate of the absolute abundance of bacteria. The drawback of this method is the sensitivity to inhibitors, competitors (human DNA) and contaminants, which compromises the reliability of the method for bacterial quantification [8]. Another limitation of this method is that measured DNA yield may be underestimated due to DNA loss at different extraction/purification steps.

Ultra-Deep Microbiome Prep (Molzym, Bremen, Germany) for the enrichment of bacterial DNA relies on the selective lysis of mammalian cells and the degradation of released DNA. This commercially available kit has proven its ability to increase the bacterial-to-human DNA ratio 3–4 log units in *fluids* such as bronchoalveolar lavage (BAL) samples [6] or uninfected sonicate fluids from prosthetic joint infections spiked with *Staphylococcus aureus* [9], allowing better assessment of the bacterial community. In another study though, the enrichment procedure did not improve pathogen-to-human DNA ratios in cerebrospinal fluid and nasopharyngeal aspirate [4].

For *tissues*, DNA extraction with the Ultra-Deep Microbiome Prep includes the proteinase K pre-treatment as an integral step aimed at dissociating the tissue to individual cells that are subsequently exposed to lysis conditions. DNA extracts from biopsies of the liver, diabetic foot, cardiovascular and orthopedic implant-adjacent tissues, obtained using the Molzym ‘tissue protocol’ had various bacterial-to-human DNA ratios, and human DNA represented the major fraction in most cases [2, 10–15]. The proteinase K treatment may cause, as an undesirable collateral effect, the lysis of certain susceptible bacteria such as those from the phyla Proteobacteria and Bacteroidetes [14, 16]. The susceptibility of bacteria to proteinase K lysis may further increase during prolonged frozen storage [16].

In the present work, we evaluated the impact of bacterial DNA enrichment on mNGS-based detection and quantification of bacterial absolute and relative abundance in infected tissue model. To mimic clinical samples of infected tissues, we spiked pig muscle tissue with four bacteria belonging to different –clinically important– phyla: Firmicutes, Proteobacteria, Bacteroidetes and Actinobacteria.

The objectives of the study were to (i) evaluate the performance of bacterial quantification in tissue by mNGS using spike-in cells, (ii) assess potential strengths and limitations of the Ultra-Deep Microbiome Prep kit (for bacterial DNA enrichment) in detecting and quantifying bacteria in tissue, and, (iii) assess the effect of replacing the original Ultra-Deep Microbiome Prep proteinase K-based tissue solubilization by a Liberase (collagenases/thermolysin) digestion/cell straining on bacterial detection and quantification.

## Material and methods

### Preparation of the pig muscle tissue

From a 3-cm thick slice of a fresh pork fillet, purchased at a local supermarket, we removed about 2-mm surface layer using a scalpel. With a new scalpel, we minced the tissue into 100–120 mg pieces, which were then frozen at −80 °C.

### Preparation of a bacterial mock community

The mock community was composed of four bacteria belonging to the four phyla commonly found in the human microbiome: *Escherichia coli* DH5-α (Proteobacteria), *Bacillus spizizenii* W23 (Firmicutes), and two clinical isolates from our strain collection—*Rothia dentocariosa* GRL-111979 (Actinobacteria) and *Sphingobacterium* sp. GRL-24 (Bacteroidetes). Bacteria were maintained on Columbia Agar plates with 5% sheep blood (bioMérieux, Marcy-l'Étoile, France) at 37 °C. The 16-h culture, grown in Difco LB Broth (Miller) (Becton, Dickinson and Company, Franklin Lakes, NJ, USA) at 37 °C with agitation at 180 rpm, was diluted with 0.9% NaCl to a McFarland turbidity of 0.5.

Each bacterial suspension was then (i) plated on blood agar to determine CFU counts after a 20-h growth and (ii) counted microscopically in a Neubauer improved (Petroff) 0.01-mm chamber (Paul Marienfeld, Lauda-Königshofen, Germany). During microscopic enumeration, bacterial suspensions in 0.9% NaCl were kept on ice. Based on microscopic enumeration results, suspensions were adjusted with 0.9% NaCl to 1×10^7^ bacteria/mL and mixed in equal volumes. The obtained mock community was divided into two parts one of which was supplemented with 1/10 volume of 99.8% glycerol. Glycerol-supplemented and non-supplemented mock communities were spiked immediately (designated *Fresh Gro* and *Fresh*, respectively), or after a 10-day frozen storage at −80 °C (*−80° Gro* and *−80°*, respectively), onto pig tissue homogenate aliquots (see below).

### Spiking of the pig muscle tissue with the mock community

On the day of spiking/DNA extraction, tissue pieces were defrosted on ice, pooled by 6 in a Microbial DNA Free 2-mL tube with 2.8 mm ceramic beads (Omni International, Kennesaw, GA, US) and ground on a Bead Ruptor 4 (Omni International) for 30 s at speed 3. Such obtained homogenates were pooled by two and then divided into 100-mg aliquots, which were spiked with the mock community containing 1.4 × 10^6^ of bacterial cells (3.5 × 10^5^ cells of each species). For that purpose, we used 140 µL of the fresh bacterial pool (*Fresh*), 154 µL of the glycerol-supplemented fresh bacterial pool (*Fresh Gro*), 140 µL of the frozen bacterial pool (*−80°*) and 154 µL of the glycerol-supplemented frozen bacterial pool (*−80° Gro*), respectively, per tissue homogenate aliquot. Each mock community stored under specified condition (*Fresh*, *Fresh Gro*, *−80°* or *−80° Gro*) was spiked onto 6 tissue aliquots (24 spiked samples in total).

### DNA extraction from the spiked pig tissue

Each of the four sets of 6 pig tissue homogenate aliquots spiked with the mock community stored under given condition was subjected to three different DNA extraction protocols: *NS-MAG*, *MOLZ* and *MOLZ-F*, using two aliquots with each method.

(1) *NS-MAG* protocol. We mixed the spiked tissue with 500 µL of GT buffer (RBC Bioscience, New Taipei City, Taiwan) in a Nucleospin Bead Tube Type A (Macherey-Nagel, Düren, Germany) containing 0.6–0.8 mm ceramic beads. The mixture was shaken on a Vortex-Genie 2 with a horizontal tube holder (Scientific Industries, Bohemia, NY, USA) at maximum speed for 20 min and further processed on a MagCore HF16 Automated Nucleic Acid Extractor (RBC Bioscience) as described previously (Lazarevic et al., 2018). DNA was eluted in 100 μL of Tris–HCl (10 mM, pH 8).

(2) *MOLZ* protocol. DNA was extracted from the spiked tissue using the Ultra-Deep Microbiome Prep kit (Molzym, Bremen, Germany) according to the manufacturer’s instructions for tissues and eluted in 100 μL of ddH_2_O. In this protocol, mammalian (originally human) tissue is solubilized using proteinase K. Bacterial DNA extraction is performed following ‘selective’ lysis of mammalian cells and endonuclease digestion of the released (and any other accessible) DNA.

(3) *MOLZ-F* protocol. We replaced the original proteinase K pre-treatment step in the *MOLZ* protocol for tissues by the addition to the spiked sample of an equal volume of 10 mg/mL Liberase TL (Roche, Basel, Switzerland) dissolved in Dulbecco’s phosphate buffered saline with MgCl_2_/CaCl_2_ (Sigma-Aldrich, Saint Louis, MO, USA). Liberase TL contains clostridial collagenases I and II and a low concentration thermolysin, a non-clostridial neutral protease. Following a 90-min digestion at 37 °C with shaking at 1 000 rpm, the samples were passed through a 100-µm cell strainer (Corning, Corning, NY, USA) which was then washed twice with 200 µL SU buffer (from Ultra-Deep Microbiome Prep). From this point we followed the Ultra-Deep Microbiome Prep protocol and eluted purified DNA in 100 μL of ddH_2_O. The purified DNA was stored at *−*20 °C.

Prior to DNA extraction using *NS-MAG* and *MOLZ* protocols, the tubes with spiked tissue homogenates were exposed to a 90-min incubation at 37 °C with shaking at 1 000 rpm. This extra incubation step (without Liberase) was introduced to adjust for the potential bacterial DNA synthesis that might occur in the *MOLZ-F* protocol during the 90-min Liberase digestion at 37 °C (see above).

### Quantification of porcine DNA

The concentration of porcine DNA was determined by qPCR targeting the beta-actin gene on a CFX96 qPCR system (Bio-Rad, Hercules, California, USA). The assay was performed in 20 µL of Absolute qPCR Mix (Thermo Fisher Scientific, Waltham, MA, USA) containing 300 nM forward (Sus_ACTB-F) primer, 300 nM reverse (Sus_ACTB-R) primer, 200 nM 5′-FAM 3′-TAMRA labeled probe (Sus1) [17] and 1 µL of DNA extract. The amplification parameters were as follows: 95 °C/15 min, followed by 42 cycles of [95 °C/15 s; 60 °C/60 s]. The reference curve was generated using known concentrations of pig genomic DNA (Sigma-Aldrich, Saint Louis, MO, USA).

### Bacterial DNA quantification

The bacterial 16S rRNA gene copy number in DNA extracts was determined by qPCR as described previously [18] using *E*. *coli* DH5-α DNA to construct a reference curve. The number of 16S rRNA gene copies was calculated considering that 1 pg of *E*. *coli* DH5-α DNA corresponds to 1493 copies of the 16S rRNA. For *NS-MAG* extracts, a correction was made for volume loss during DNA extraction.

### mNGS

Sequencing libraries were prepared using a Nextera DNA Flex Library Prep kit (Illumina, San Diego, CA, USA) with 6 ng of input DNA and 12 amplification cycles. Paired-end sequencing (2×151) was performed on an iSeq 100 System (Illumina). In each run, six 0.02 nM libraries were multiplexed and spiked with PhiX control at 1%.

### Initial processing of mNGS reads

Sequencing reads were quality filtered with Trimmomatic v0.36 (SLIDINGWINDOW:10:30 MINLEN:100). Replicate read pairs were removed with a home-made script (available at https://github.com/GRL-HUG/duplicates_2). Reads pairs matching the pig (*Sus scrofa*) genome (GenBank accession GCA_000003025) were identified using CLARK v.1.2.6.1 (m -0) [19] and filtered out. Remaining read pairs were aligned to genomes of the mock-community members or processed using the metagenomic pipeline.

### Alignment of mNGS read to genomes of the mock-community strains

After initial processing of mNGS reads described above, mapping of the forward and reverse reads to the draft genome sequences of the four mock-community members was performed using USEARCH [20] (-usearch global -strands both -id 0.9 -query_cov 0.9 -evalue 1e-10 -top_hit_only -wordlength 25).

### Metagenomic pipeline

Following the initial processing of mNGS reads (see above), we performed an additional removal of pig-related reads by BWA-mem [21]. Low-complexity reads were removed using Komplexity (–threshold 0.5 –filter) [22]. Filtered read pairs were sequentially classified against three custom databases (Supplementary Table 1) of Latest RefSeq genome assemblies [23] (downloaded on 17 February 2022) using Kraken 2 (–confidence 0.05); [24] the reads that remained unclassified after a given round of the analysis were used as input for the next one. The three custom databases were respectively built of: (i) bacterial reference, representative and completely sequenced genomes and Bacteria Candidate Phyla representative genomes (*n* = 33 000); (ii) archaeal reference, representative and completely sequenced genomes (*n* = 599), fungal reference and representative genomes (*n* = 420), representative genomes of human protozoan parasites (*n* = 21; taxa names were compiled from https://wikipedia.org/wiki/List_of_parasites_of_humans) and genomes of DNA viruses from the genera that infect humans (taxa names were compiled from viralzone.expasy.org) (*n* = 634); and (iii) genomes of DNA bacteriophages and archaeal viruses (taxa names were compiled from viralzone.expasy.org) (*n* = 4 588). From the Kraken 2 outputs, we re-estimated species abundance using Bracken [25].

### DNA extraction, genome sequencing and assembly of spike-in strains

Strains of *E*. *coli, B. spizizenii*, *R*. *dentocariosa* and *Sphingobacterium* sp. were grown overnight on Columbia Agar with 5% sheep blood plates (bioMérieux) at 37 °C. Several colonies were harvested and suspended in 500 µL of GT buffer. DNA was extracted using the *NS-MAG* protocol as described above. Sequencing libraries were prepared using a Nextera DNA Flex Library Prep kit with 100 ng of input DNA and 5 amplification cycles. The four 0.02 nM libraries were pooled and spiked with 1% PhiX control. Paired-end sequencing (2 × 151) was performed on an iSeq 100 System.

Sequencing reads were quality filtered with Trimmomatic v0.36 (SLIDINGWINDOW:10:30 MINLEN:100) [26]. To remove putative contaminant reads, we performed two filtering steps. In the first, we filtered out all read pairs that matched NCBI reference human genome sequence (GRCh38.p13) using CLARK (-m 0). Remaining read pairs were then classified using CLARK (-m 0 -c 0.8) against the bacterial database (see above and Supplementary Table 1). For each of the 4 sequenced bacterial species, the reads pairs assigned to a class other than that to which the given species belongs, were removed. Filtered read pairs were assembled with SPAdes v3.12.0 [27] followed by QUAST v5.0.2 [28] evaluation. The draft genomic sequences used as a reference for read mapping (see below) included all contigs >500 nt and the sum of their nucleotides was used as proxy for genome size.

Average nucleotide identity (ANI) between the genome sequence assembly of *Sphingobacterium* sp. GRL-24 and type strains of *Sphingobacterium* species from the NCBI Assembly database [23, 29] was calculated using *pyani* [30] with BLAST [31] method. When compared to type strains of *Sphingobacterium* species, the strain GRL-24 showed the best ANI to *Sphingobacterium siyangense* (96.1%) followed by *Sphingobacterium multivorum* (91.5%). We therefore considered the strain GRL-24 as belonging to *S. siyangense*.

## Results

### Quantification of pig (‘host’) and bacterial loads by qPCR

**Fig. 1:**
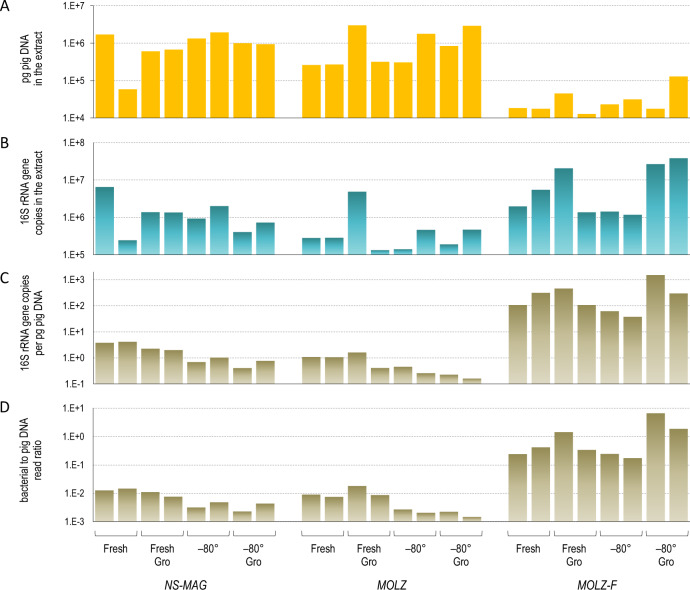
Quantification of pig and bacterial DNA in *NS-MAG*, *MOLZ* and *MOLZ-F* extracts obtained from samples following four storage conditions. **A** Pig DNA quantified by qPCR. **B** Bacterial 16S rRNA gene copy number quantified by qPCR. **C** Bacterial 16S rRNA gene copy number (as in **B**) normalized per pig DNA mass (as in **A**). **D** Ratio of bacterial-to-pig sequencing reads in the mNGS dataset. The number of read pairs corresponding to pig genome was identified by CLARK. For bacteria, we calculated the average for the number of forward and reverse reads mapped by USEARCH to genome sequence assemblies of the four mock-community species.

For all four tested storage conditions (*Fresh*, *Fresh Gro*, *−80°* or *−80° Gro*) of the mock community, qPCR revealed the lowest levels of extracted pig (‘host’) DNA for the *MOLZ-F* bacterial enrichment protocol (Fig. 1A). Bacterial 16S rRNA gene copy number qPCR estimates were higher in *MOLZ-F* extracts than in those obtained with the original *MOLZ* protocol (Fig. 1B). As a consequence, the counts of bacterial 16S rRNA gene copies, normalized per host DNA mass, were considerably higher (1–3 log10 units) for the *MOLZ-F* in comparison to the two other extraction methods (Fig. 1C).

### Bacterial-to-‘host’ DNA ratio assessed by mNGS

In line with the above observations, the analysis of mNGS data revealed more than two orders of magnitude higher bacterial-to-host reads ratio in *MOLZ-F* than in *MOLZ* or *NS-MAG* extracts (Fig. 1D). Moreover, the qPCR (Fig. 1C) and mNGS-determined (Fig. 1D) bacterial-to-host DNA ratio estimates profiles were highly congruent.

**Fig. 2:**
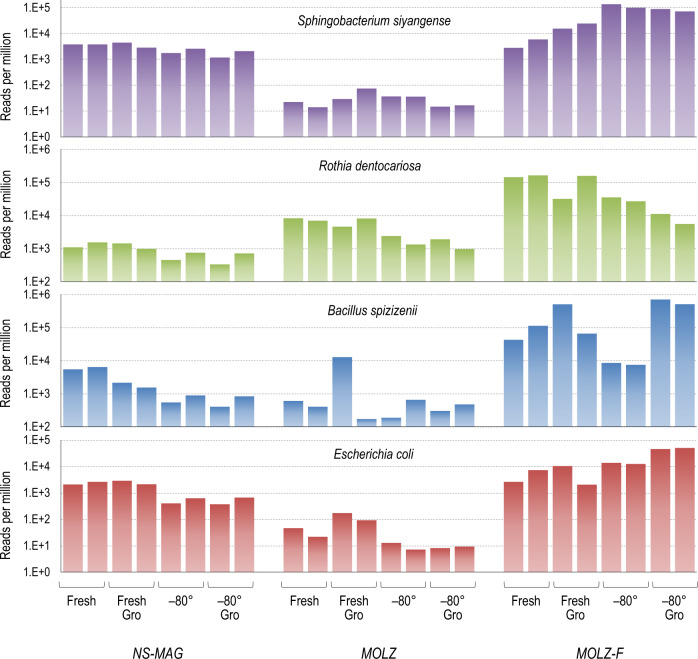
Read counts of the four spike-in organisms normalized per million of quality filtered read pairs. For each bacterial species, we calculated the average for the number of forward and reverse reads mapping by USEARCH to relevant genome sequence assembly.

For each of the four mock-community species we also calculated bacterial read counts normalized per million total quality filtered reads. Overall, the *MOLZ-F* protocol performed the best for all of the tested species, generating substantially higher normalized bacterial counts compared to standard enrichment (*MOLZ*) or no-enrichment (*NS-MAG*) procedures. In *MOLZ* samples, the normalized read counts of gram-negative bacteria (*E*. *coli* and *S*. *siyangense*) were noticeably lower than in *MOLZ-F* and *NS-MAG* samples (Fig. 2), while the gram-positive *R*. *dentocariosa* levels were intermediate between those of *MOLZ-F* and *NS-MAG* treated samples (Fig. 2).

### Taxonomic profiling of the mock community

**Fig. 3:**
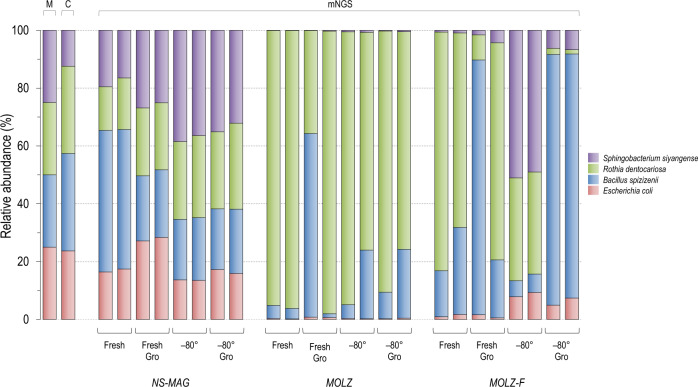
Relative abundance of the four bacterial species in the mock-community determined by direct microscopic examination, CFU counts and mapping of mNGS reads to genomic sequences of the four mock-community members. Microscopic cell counting and plating for CFU enumeration were determined separately for each species, prior to the creation of the mock community. In the mNGS analysis, porcine sequences were removed by CLARK to generate the input dataset. Reads were then aligned by USEARCH to the genomic sequences of the four mock-community members and the correction for genome size was performed. M, direct microscopic examination; C, CFU (colony forming unit).

To infer the relative abundance of the four mock-community species using mNGS, we aligned their sequencing reads to corresponding genome assemblies and performed correction for differences in genome sizes. The obtained mNGS taxonomic profiles of unenriched samples were consistent with those of microscopic and culture-based enumerations. However, the proportions of the four spiked bacterial species in mNGS datasets of enriched samples were distorted relative to the actual (i.e., spiked amount) ratios (Fig. 3). In particular, the standard enrichment procedure was associated with marked dominance of gram-positive bacteria. The modified enrichment protocol (*MOLZ-F*), when compared to standard enrichment, generated taxonomic profiles closer to the actual ones for all tested storage conditions; this effect was more pronounced for frozen samples, particularly those stored without glycerol.

We also performed taxonomic profiling of the mock community using Kraken 2, a common fast-processing tool for classification of millions of mNGS reads. In this metagenomic pipeline, we used k-mer databases constructed from thousands of genomes of different organisms, to mimic a standard situation when analyzing samples of unknown microbial composition. The obtained ratios among the spiked species were similar to those found in the alignment-based approach (Fig. 3) but some drawbacks of the k-mer based fast-processing were identified. A substantial fraction of reads in *NS-MAG* and *MOLZ* samples was assigned to species different from those that constituted the mock community, notably *Nesterenkonia natronophila* (phylum Actinobacteria) (Supplementary Fig. 1). Closer inspection of these reads revealed their origin from porcine satellite DNA that were not filtered out during the *in silico* removal of host-derived reads, a situation resembling recently reported misclassification of bovine satellite DNA as mycobacterial [32].

### Spike-in-based mNGS quantification of the bacterial load

We evaluated the performance of bacterial load quantification considering individually each of the four spike-in species as calibrator and the other co-spiked species as test organisms. This quantification test takes into account (i) the actual load of the calibrator organism spiked into the sample prior to DNA extraction, determined by microscopic enumeration, and (ii) mNGS read counts assigned to this calibrator and tested co-spiked organisms, with corrections for genome size.

**Fig. 4:**
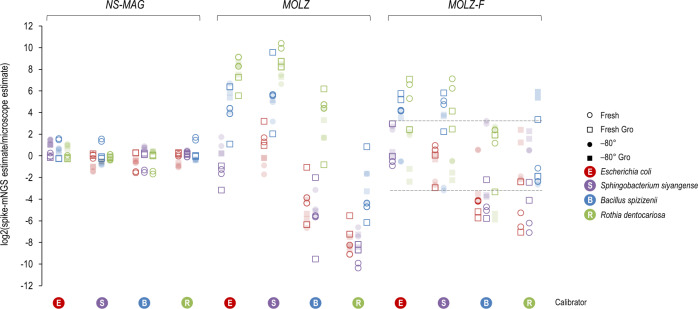
log2-transformed ratio between spike-based mNGS and microscopic quantification of the mock-community members. The mock-community member used as a calibrator to calculate the absolute abundance of the three other co-spiked organisms is marked on the bottom line. Dashed lines denote the range of values obtained for samples stored at –80 °C without glycerol and processed using the *MOLZ-F* method.

We determined the ratio between the measured mNGS calibrator-based bacterial load and actual (microscopy-based) load for all calibrator-test organism combinations across different mock-community storage conditions and DNA extraction methods (Fig. 4). In unenriched (*NS-MAG*) samples, the measured load deviated little from the actual load across all tests. Differences between measured and actual loads were marked for the original enrichment protocol. In these cases, the measured loads of gram-positive bacteria were overestimated using gram-negative bacteria as calibrators, whilst the abundance of gram-negative bacteria was underestimated when gram-positive bacteria were considered calibrators. Using the modified enrichment protocol (*MOLZ-F*), the ranges of deviation of measured mNGS calibrator-based loads from the actual ones were narrower as compared to the original *MOLZ* protocol. In particular, for frozen samples without glycerol, calculated absolute abundances differed from the actual ones up to 9.3-fold and up to 326-fold in *MOLZ-F* and *MOLZ* samples, respectively.

## Discussion

The ability of mNGS to detect and quantify bacteria in tissues depends on a number of factors such as the bacterial load, the ratio of bacterial to human cells (and thus DNA), the sequencing depth and the use of bacterial DNA enrichment. In this study we showed both the advantages and drawbacks of bacterial DNA enrichment based on the selective lysis of host tissue cells and subsequent removal of accessible DNA by DNase.

In unenriched DNA extracts of spiked tissues, obtained following combined mechanical/chemical cell disruption (*NS-MAG*), estimates of the relative and absolute abundances of bacteria were fairly accurate. An obvious limitation of this approach is that low-abundance bacteria may remain unidentified due to overwhelming ‘host’ reads.

Pathogen load is one of criteria used for distinguishing between colonization and infection although there is no universal cut-off value for that purpose. For example, for human BAL fluid, 10^4^ bacteria/mL is most often used as a threshold for infection, but higher and lower positivity thresholds have been reported [33]. Similarly, a bioburden of 10^5^ bacteria/g tissue is used as an arbitrary threshold for wound infection, although lower counts of more virulent bacteria may be associated with infection [34, 35]. Based on the results obtained with unenriched samples (*NS-MAG*), we estimate that for 10^5^ bacteria per g of tissue, about 60 reads per million total reads would originate from bacteria. Therefore, a bacterial burden in the range of 10^3^–10^4^ per g of tissue would generate number of reads too close to the limit of detection to allow reliable interpretation. For a more reliable detection of low-abundance bacteria, there are two strategic options: (i) deeper sequencing, which is associated with increased costs and negative environmental impact due to higher data production and storage [36] and (ii) enrichment of bacterial DNA by removing host DNA prior to sequencing.

The bacterial DNA enrichment with a standard *MOLZ* protocol compromised the detection of gram-negative bacteria. Taxonomic profiles of the mock community were substantially distorted due to depletion of DNA from gram-negative bacteria and consequent overestimated relative abundance of gram-positive ones. Likewise, the choice of the calibrator species impacted the results of the absolute quantification; the use of gram-negative bacteria as calibrators inflated the absolute abundance estimates of gram-positive bacteria, while measured loads of gram-negative species were underestimated when gram-positive bacteria were used as calibrators.

The substitution of the original proteinase K tissue solubilization step of the *MOLZ* protocol by a Liberase treatment and cell straining (*MOLZ-F*) substantially improved mNGS bacterial *detection*, i.e., increased the ratio of bacterial-to-host reads for each of the four tested species across all the four mock-community storage conditions. In addition, compared to the original enrichment protocol, the modified enrichment procedure was associated with less distorted cell counts estimates (relative to actual values). The best performance of bacterial absolute abundance measurements among eight tested combinations of enrichment (*n* = 2) and mock-community storage conditions (*n* = 4) was observed using the modified enrichment procedure and the mock-community frozen without glycerol. In this particular case, estimated loads were less than one log10 unit different from actual ones. Hypothetically, storage conditions may differentially affect (i) susceptibility/resistance of different bacterial species to lysis during the stress conditions aimed at disrupting host tissue, host cells and bacterial cells and (ii) DNA synthesis in different bacterial species during the 90-min incubation of spiked tissue with Liberase at 37 °C.

Modified enrichment procedure could be applied to virtually any tissue suspected of infection in order to identify infection-causative agent(s). Of particular interest are tissues known to be often infected by slow growing and fastidious bacteria, such heart valves, bones and joints but also abscesses at different body sites (liver, brain and lymph nodes). Bacteria in viable but not culturable state induced by antibiotic treatment could also be detected. Of course, the superiority of the modified (*MOLZ-F*) over original (*MOLZ*) enrichment protocol will have to be tested on a larger panel of spike-in bacteria and tissue specimens with different consistencies, bacterial loads, host-to-bacterial cell ratios and storage conditions. Here we focused on bacteria that belong to the four phyla (Firmicutes, Proteobacteria, Bacteroidetes and Actinobacteria) commonly seen across different body sites. Investigation of the clinically relevant species from other phyla, such as *Mycoplasma pneumoniae* (phylum Tenericutes) and *Fusobacterium nucleatum* (phylum Fusobacteria) might reveal additional interesting observations. The ability of bacteria to resist the effects of the bacterial DNA enrichment procedure may vary even within closely related species. For example, variations in the peptide moiety of the bacterial cell wall peptidoglycan, observed even within the same genus [37], can make a species more or less susceptible to lysis by proteinase K [38] during tissue solubilization.

**Fig. 5:**
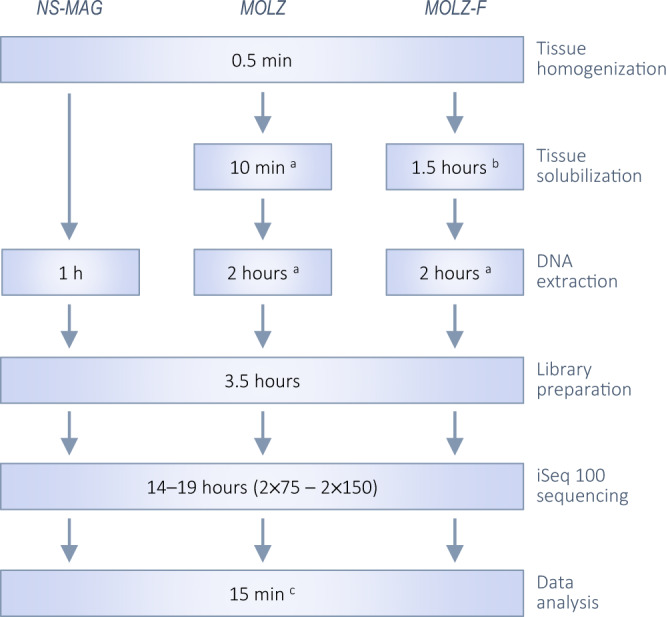
Timing for different steps of the mNGS analysis. ^a^Carried out with the Ultra-Deep Microbiome Prep (Molzym) kit and protocol for tissues. ^b^Replacement of the proteinase K digestion by Liberase treatment and cell straining. ^c^The estimate for an alpha version of our automated Kraken 2-based analysis for bacterial quantification of spiked-in samples. An additional 1.5-h incubation (without Liberase), introduced in this study in the *NS-MAG* and *MOLZ* procedures to adjust for the potential effect of bacterial DNA synthesis (that might occur in *MOLZ-F* during the 1.5-h tissue solubilization step), is not presented.

DNA extraction with the modified enrichment protocol (*MOLZ-F*) requires an additional 2.5 h compared to the no-enrichment *NS*-*MAG* protocol. However, in relative terms, this only leads to a moderate increase of the overall turnaround time, from ∼24.5 to ∼27 h (Fig. 5). With a 2 × 75 instead of 2 × 150 iSeq 100 sequencing mode, the whole process could be completed in ∼22 h. Further reduction of the overall turnaround time to ∼8–10 h (including the modified enrichment procedure) could be achieved using Oxford Nanopore Technologies sequencing [39].

The organism(s) used as calibrators for bacterial quantification should not be members of the host microbiome. Environmental and plant-associated species showed suitability for calibrating the ratios of absolute abundances of endogenous bacteria in metataxonomic (16S rRNA gene based) analyses of unenriched stool samples [40] and metagenomic analysis of liquid (BAL) samples [41]. From a practical point of view, frozen aliquots of a calibrator suspension are convenient as they can be used over longer times than freshly prepared ones. The compatibility of commercially available frozen spike-in cell mixtures with bacterial DNA enrichment protocols should be tested because the stabilization agents potentially present in these formulations may change cell integrity and increase the susceptibility to DNA digestion during the host DNA removal. Freeze-dried commercial preparations of spike-in bacteria also may have altered susceptibility/resistance to cell lysis. Lastly, the use of spike-in DNA as calibrators for bacterial quantification [42, 43] is not compatible with enrichment procedures that include DNase treatment for host DNA removal, unless the spike is added after the DNase inactivation step.

The use of reliable bioinformatics pipelines has been recognized as a challenge in clinical metagenomics but the exploration of this topic was beyond the scope of our study. We aligned sequencing reads to genomic sequences of the four relevant strains (which were sequenced for the purpose of this study) using stringent (USEARCH) matching parameters. However, such an approach is not applicable for the first-line analysis of clinical samples because the infectious agent(s) they contain are not known. Usually, a k-mer-based analysis is used, which allows fast classification of millions of sequencing reads against a large reference database. The k-mer-based Kraken analysis, which we also performed, revealed a small proportion of reads corresponding to organisms different from those of the mock-community used. This points to the importance of performing ‘blank’ extraction controls (with no biological material or with a ‘healthy’ tissue) to define background [39] and artifactual [32] organisms specific to the pipeline in place in each given laboratory.

In the present study, we used a defined system consisting of pig tissue spiked with relatively high bacterial loads, which made the control for reagent and other laboratory contaminants less important than when analyzing specimens with low microbial biomass [5]. In real clinical tests, the addition of the same amount of the calibrator bacteria in the clinical specimen and in corresponding blank sample would allow better distinction between contaminants and truly present microorganisms by the subsequent mNGS-based quantification. This also applies to situations with an incidental cross-contamination of the control sample by small amount of the clinical specimen; in this case, the absolute abundances are clearly more informative than the relative ones.

Having an unbiased method for bacterial DNA enrichment would ultimately accommodate both high detection sensitivity and accurate quantification of bacteria in tissue by mNGS. This would eventually also resolve current limitations of the culture in bacterial detection and quantification beyond poor or no growth, such as formation of chains, clumps or biofilms. In the meantime, processing of a tissue sample in parallel with and without enrichment could be envisaged. mNGS of unenriched samples would accurately quantify high-burden bacteria. The modified enrichment protocol would increase the likelihood of bacterial detection and, by virtue of higher genome coverage, improve in silico typing and prediction of antibiotic resistance.

## Supplementary information

Supplementary Materials

Supplementary Tables

## Data Availability

The sequence datasets presented in this study can be found in European Nucleotide Archive (ENA; https://www.ebi.ac.uk/ena) under study number PRJEB47877.
