# Supplementary material for: Effect of bacterial DNA enrichment on detection and quantification of bacteria in an infected tissue model by metagenomic next-generation sequencing
Source: ISME Commun. 2022 Dec 26;2:122. doi: 10.1038/s43705-022-00208-2 (PMC9792467; doi:10.1038/s43705-022-00208-2)

Supplementary Material

Effect of bacterial DNA enrichment on detection and quantification of bacteria in an infected tissue model by metagenomic next-generation sequencing

Authors: Vladimir Lazarevic, Nadia Gaïa, Myriam Girard, Florian Mauffrey, Etienne Ruppé, Jacques Schrenzel

Supplementary Figure 1. Taxonomic profiles generated using a metagenomic pipeline. Porcine sequences were removed by CLARK and BWA to generate the input data set. The relative abundances of sequence reads were determined by Kraken 2 using a k-mer database generated from bacterial, archaeal, fungal, protozoan, viral and phage genomic sequences. No correction for genome size was performed. M, direct microscopic examination; C, CFU (colony forming unit); Gro, glycerol; mNGS, metagenomic next-generation sequencing.

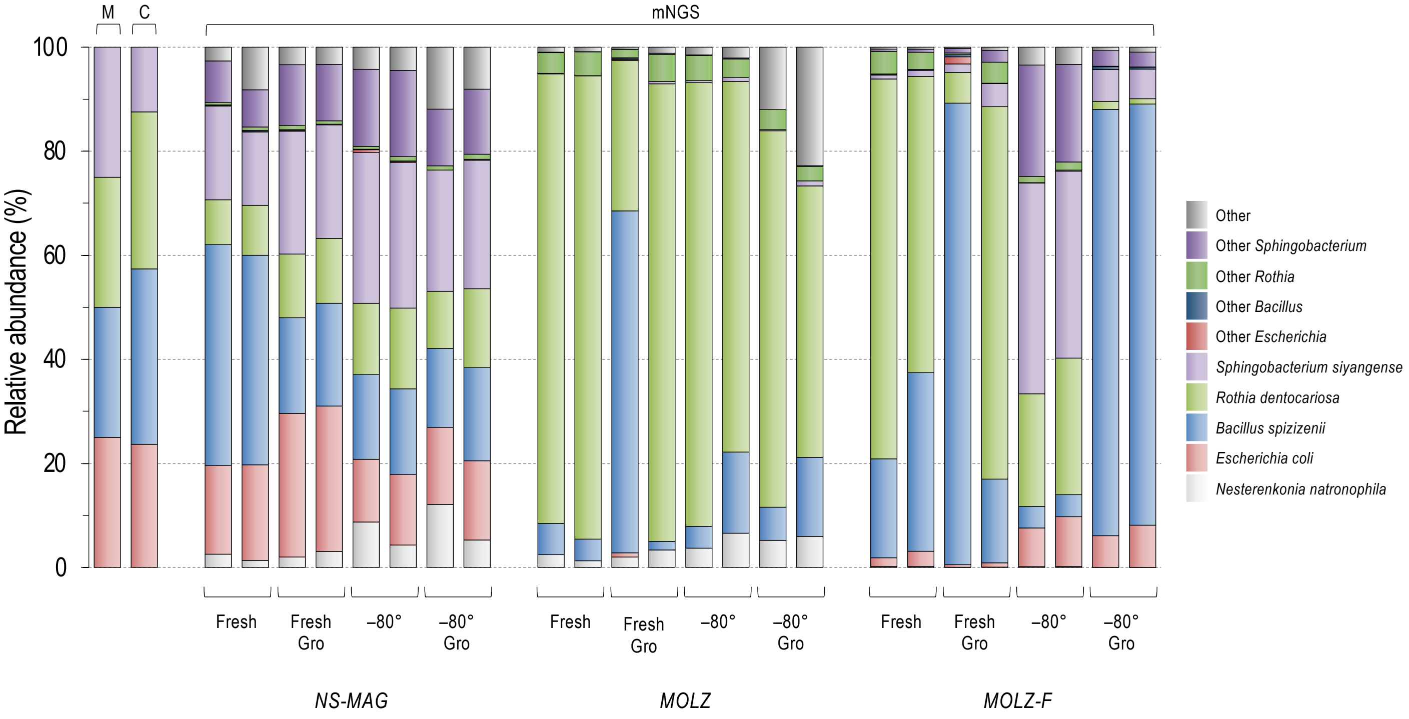

Supplement: Supplementary file 1 — Supplementary Materials [file 43705_2022_208_MOESM1_ESM.pdf]
